# Gap locations influence the release of carbon, nitrogen and phosphorus in two shrub foliar litter in an alpine fir forest

**DOI:** 10.1038/srep22014

**Published:** 2016-02-24

**Authors:** Wei He, Fuzhong Wu, Wanqin Yang, Danju Zhang, Zhenfeng Xu, Bo Tan, Yeyi Zhao, Meta Francis Justine

**Affiliations:** 1 Long-term Research Station of Alpine Forest Ecosystems, Institute of Ecology & Forestry, Sichuan Agricultural University, Chengdu 611130, China; 2 Collaborative Innovation Center of Ecological Security in the Upper Reaches of Yangtze River, Chengdu 611130, China

## Abstract

Gap formation favors the growth of understory plants and affects the decomposition process of plant debris inside and outside of gaps. Little information is available regarding how bioelement release from shrub litter is affected by gap formation during critical periods. The release of carbon (C), nitrogen (N), and phosphorus (P) in the foliar litter of *Fargesia nitida* and *Salix paraplesia* in response to gap locations was determined in an alpine forest of the eastern Qinghai-Tibet Plateau via a 2-year litter decomposition experiment. The daily release rates of C, N, and P increased from the closed canopy to the gap centers during the two winters, the two later growing seasons and the entire 2 years, whereas this trend was reversed during the two early growing seasons. The pairwise ratios among C, N, and P converged as the litter decomposition proceeded. Compared with the closed canopy, the gap centers displayed higher C:P and N:P ratio but a lower C:N ratio as the decomposition proceeded. Alpine forest gaps accelerate the release of C, N, and P in decomposing shrub litter, implying that reduced snow cover resulting from vanishing gaps may inhibit the release of these elements in alpine forests.

Carbon and nutrient release from shrub litter is one of the main pathways of forest material cycling and energy flow^1^. Recently, accelerating climate change^2^,^3^,^4^, frequent mountain disasters^5^, artificial disturbances and other factors^6^ may have accelerated the formation of forest gaps, which might favor the growth of shrubs and other understory plants and increase their litter production^7^,^8^. The roles and status of shrubs and their debris in forest ecosystems might become increasingly important^9^. However, the effects of forest gap formation on the functions of shrubs in material cycling are often overlooked in classical ecology.

Zhang & Zak^10^ reported that litter decomposition was faster under a closed canopy or in small gaps than in large gaps in a subtropical forest, whereas Denslow *et al.*^11^ found no relationships between gap sizes and litter decay rates in a wet tropical forest. However, our previous study in an alpine fir forest^12^ and the findings of Prescott *et al.*^13^ in cold forests of British Columbia showed that gaps accelerated litter mass loss. The effects of forest gaps on the decomposition of plant debris might be related to the climate zone. In regions with seasonal snow cover, forest gaps often create gradients of snow-cover depth in winter and temperature regime gradients in the growing season progressing from the gap’s center to the adjacent closed canopy due sheltering by the canopy^9^,^14^. The effects of alpine forest gaps on litter decomposition and nutrient release might vary with the seasons. However, the conflicting findings among these studies mean that the available results are inconclusive with regard to ways in which forest gaps control the release of carbon and nutrients in shrub litter in alpine forest ecosystems.

In high-altitude forests, thicker snow cover in a gap center serves as an insulator that can maintain a sufficiently warm temperature to support substantial biotic activity^15^, contributing to litter decomposition^16^ and nutrient release^17^,^18^ during winter. Additionally, the melting of snow cover favors leaching of soluble carbon and nutrients from litter^19^, and this release may be great during the snowmelt period^20^. Conversely, where the snow cover is thin or absent at the forest edge and below the closed canopy, litter is often exposed to extreme sub-freezing temperatures and undergoes more intense repeated freeze-thaw cycles, which can damage the physical structure of the litter and improve its decomposability^21^. A much thicker snow cover can form early on the forest floor in the center of the gap, and ablation may occur later there^22^,^23^. The diverse microenvironments produced by differing levels of interception of snowfall by the canopy during winter will thus have varying effects on the release of foliar litter carbon and nutrients. At present, little attention has been paid to the release of carbon and nutrients from shrub foliar litter driven by snow-cover gradients created by forest gaps in alpine forests.

During the growing season, in contrast, gap centers in alpine forests often exhibits a higher soil surface temperature than the gap edge and the closed canopy due to the center’s greater amount of solar irradiation. This warmer temperature might be the dominant factor that drives litter carbon and nutrients release via its indirect effects on hydrothermal dynamics and microbial activities of microenvironment^21^. Furthermore, precipitation in gap centers may have a direct effect on leaching of carbon and nutrients from litter. Nevertheless, a change in litter quality following its physical destruction during winter will contribute greatly to carbon^16^ and nutrient release^17^, particularly from litter where the snow cover is thin or absent during winter. Additionally, the crown canopy can intercept rainfall and create shelter, thus controlling transpiration and affecting temperature and moisture regimes^10^, which may also have strong effects on the release of carbon and nutrients from litter. However, these processes are not well understood. Therefore, based on our previous studies, we hypothesized that forest gaps can accelerate the release of carbon and nutrients in shrub foliar litter during the cold season, whereas the forest gap can slow this process during the growing season.

The alpine forest that is located in the upper reaches of the Yangtze River and on the eastern Tibet Plateau plays important roles in conserving headwaters and soils, nursing biodiversity and indicating climate change^24^. Shrubs account for approximately 15% of the biomass in the alpine forest ecosystems^25^. To test the hypothesis mentioned above, a field experiment was performed using litterbags containing foliar litter from two dominant understory shrubs (*Fargesia nitida* and *Salix paraplesia*). These bags were distributed from the gap centers to the closed canopy on the forest floor in three selected gaps in the alpine fir forest. The forest soil is always documented with low nitrogen and phosphorus concentrations, which limits forest productivity^24^. To understand the effects of gap locations on the release of carbon, nitrogen and phosphorus in shrub foliar litter during the cold winter and the growing season, a 2-year litter decomposition experiment was performed. Releases of carbon, nitrogen and phosphorus from shrub foliar litter were measured from the gap center to the closed canopy. The results are expected to provide insights into the roles of shrubs and forest gap formation on carbon, nitrogen and phosphorus cycles in high-altitude forest ecosystems.

## Results

### Carbon release

**Figure 1:**
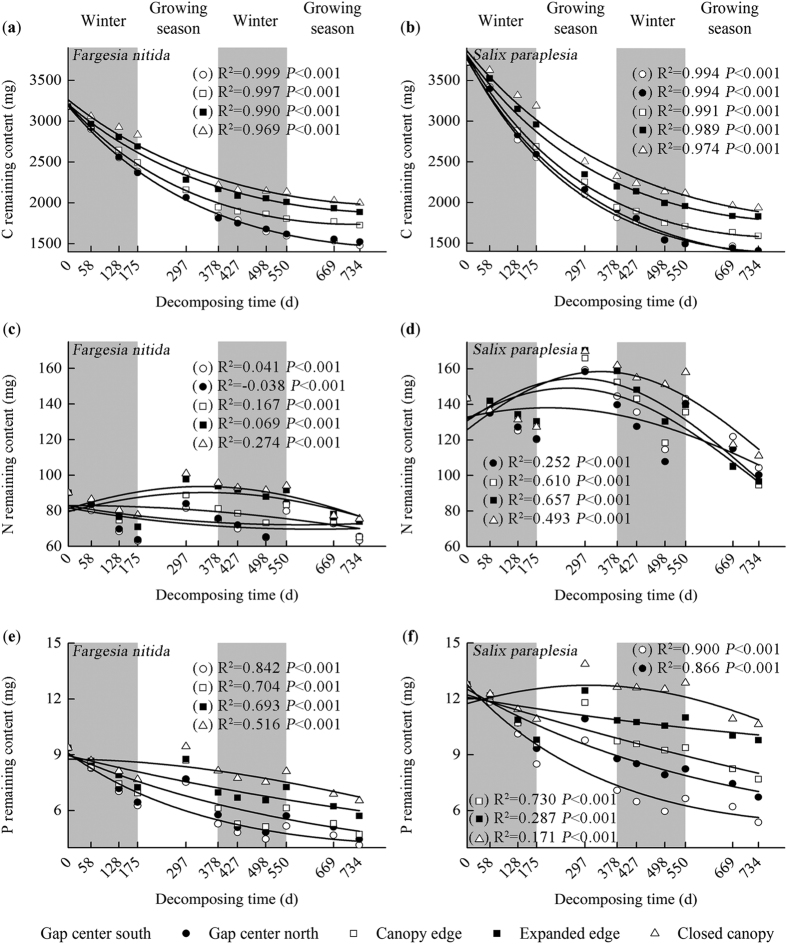
Foliar litter C (**a,b**), N (**c,d**), and P (**e,f**) remaining content in different decomposition periods across landscape locations of the forest gaps in the alpine fir forest of the eastern Qinghai-Tibet Plateau during the two-year study. Values shown are the mean ± 95% confidence intervals (n = 3). Results of two-way ANOVA (gap location was treated as a nested factor) suggest significant effects of the gap location (F_(4,100)_ = 47.008 to 939.519, *P* < 0.001, Supplementary Table S4) and the sampling period (F_(45,100)_ = 80.484 to 316.546, *P* < 0.001, Supplementary Table S4) on the remaining C, N, and P contents.

The C content decreased exponentially with time, as shown in Fig. 1a,b. There was a clear increasing tendency from the gap centers to the closed canopy during each specific period, although a significantly lower C content was observed in the willow litter than in the bamboo litter at the end of experiment. Over the 2-year decomposition period, C release from the closed canopy to the gap center south ranged from 37.19% to 53.60% in the bamboo litter and 47.84% to 62.17% in the willow litter (Fig. 1a,b). The majority (30.23% to 41.98% in the bamboo and 37.57% to 51.16% in the willow) of C release occurred during the first year of the decomposition experiment, particularly during the first winter (10.89% to 24.98% in the bamboo and 14.25% to 31.30% in the willow). During the second year of the experiment, only 6.95% to 11.62% and 9.40% to 13.47% of litter C was released from the bamboo and willow litter, respectively.

**Figure 2:**
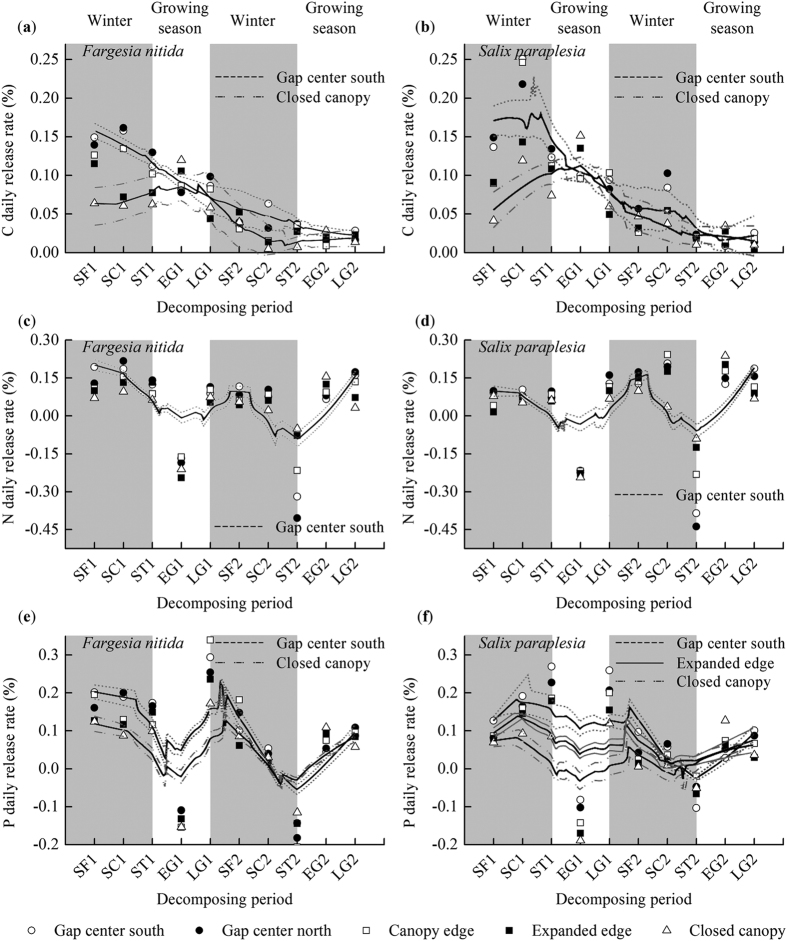
Foliar litter C (**a,b**), N (**c,d**), and P (**e,f**) daily release rates in different decomposition periods across landscape locations of the forest gaps in the alpine fir forest of the eastern Qinghai-Tibet Plateau during the two-year study. Values shown are the mean ± 95% confidence intervals (n = 3). Results of two-way ANOVA (gap location was treated as a nested factor) suggest significant effects of gap location (F_(4,100)_ = 3.605 to 20.597, *P* < 0.01, Supplementary Table S5) and sampling period (F_(45,100)_ = 17.911 to 244.904, *P* < 0.001, Supplementary Table S5) on the C, N, and P daily release rates. SF1, the first snow-formation period; SC1, the first snow-cover period; ST1, the first snow-melting period; EG1, the first early growing season; LG1, the first later growing season; SF2, the second snow-formation period; SC2, the second snow-cover period; ST2, the second snow-melting period; EG2, the second early growing season; LG2, the second later growing season.

The *V*_*L*_ of C associated with both species showed a decreasing tendency from the gap center to the closed canopy during the first snow-formation period and the two snow-cover periods, snow-melting periods and later growing seasons, whereas the opposite trend was observed during the two early growing seasons (Fig. 2a,b). Among the ten periods and for both species, the highest *V*_*L*_ of C was observed in the gap centers and below the canopy edge during the first snow-cover period, and the highest *V*_*L*_ of C was observed below the closed canopy during the first early growing season (Fig. 2a,b).

### Nitrogen release

During the overall experimental period, the N release from the bamboo litter was 16.32% to 29.76% from the closed canopy to the gap centers, and that of the willow litter was 22.48% to 34.01% (Fig. 1c,d). Although N release was observed in both types during the first winter, the release from the bamboo and willow litter was only −5.94% to 16.86% and −13.01% to 2.47% during the first year and 11.23% to 22.26% and 28.09% to 43.34% during the second year, respectively. The gap centers displayed higher and lower N enrichment during the periods of days 498–550 and days 175–297, respectively.

No significant differences in the responses of the *V*_*L*_ of N in both litter species based on decomposition period were found for the two species among the gap locations (Fig. 2c,d). Compared to the expanded edge and the closed canopy, the two litter types in the gap centers displayed higher *V*_*L*_ of N during the first snow-melting period and the two snow-formation periods, snow-cover periods, and later growing seasons but lower rates during the second early growing season. The highest *V*_*L*_ of N in the gap centers and canopy edge was observed in the bamboo during the first snow-cover period and in the willow litter during the second snow-cover period, and the highest values in both types below the closed canopy were observed during the second early growing season (Fig. 2c,d).

### Phosphorus release

Over the two-year experiment, the P release from the bamboo and willow increased from 30.15% to 55.77% and 16.85% to 57.98% from the closed canopy to the gap center, respectively (Fig. 1e,f). During the first year, 13.11% to 43.54% of the P was released from the bamboo, and 1.14% to 44.51% was released from the willow. During the second year, 12.23% to 17.04% of the P in the bamboo and 8.32% to 16.13% in the willow were released. There was an absolute release of P from both litter types during the first three periods (the first winter), and the highest P release from both types among the ten periods in all locations occurred during the first later growing season (the period of days 297–378).

Compared with the expanded edge and closed canopy, the two litter types in the gap centers displayed higher *V*_*L*_of P during the two snow-formation periods, snow-cover periods, and later growing seasons and during the first snow-melt period but lower *V*_*L*_ of P during the second early growing season. Moreover, the gap centers displayed higher and lower P enrichment during the second snow-melting period and the first early growing season, respectively. Over the ten periods, the highest *V*_*L*_of P from both litter types in all locations occurred during the first later growing season, with the exception of the willow litter, which displayed the highest *V*_*L*_of P in the gap centers and below the expanded canopy during the first snow-melting period and below the closed canopy during the second early growing season (Fig. 2e,f).

### Stoichiometric ratios

**Figure 3:**
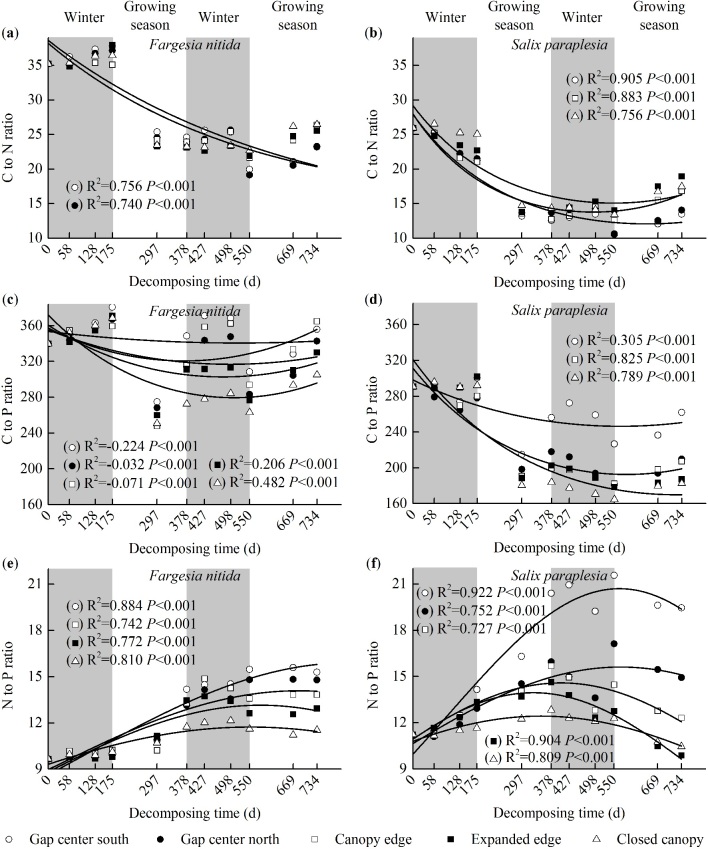
Exponential regression of foliar litter C:N (**a,b**), C:P (**c,d**) and N:P (**e,f**) ratios to decomposition time across landscape locations of the forest gaps in the alpine fir forest of the eastern Qinghai-Tibet Plateau during the two-year decomposition period. Values shown are the mean (n = 3). Results of two-way ANOVA (gap location was treated as a nested factor) suggest significant effects of gap location (F_(4,100)_ = 7.914 to 1121.545, *P* < 0.01, Supplementary Table S6) and sampling period (F_(45,100)_ = 64.239 to 428.597, *P* < 0.001, Supplementary Table S6) on the pairwise ratios among C, N, and P.

The C:N and C:P ratios decreased and the N:P ratios increased as the decomposition proceeded (Fig. 3). There was an increase in the C:N ratio in the bamboo litter from the gap center to the closed canopy after day 550 and in the willow litter during each experimental period (Fig. 3a,b). In contrast, the ratios of C:P and N:P decreased from the gap center to the closed canopy as the decomposition proceeded (Fig. 3c–f).

## Discussion

### Carbon, nitrogen and phosphorus release across gap locations in winter

**Figure 4:**
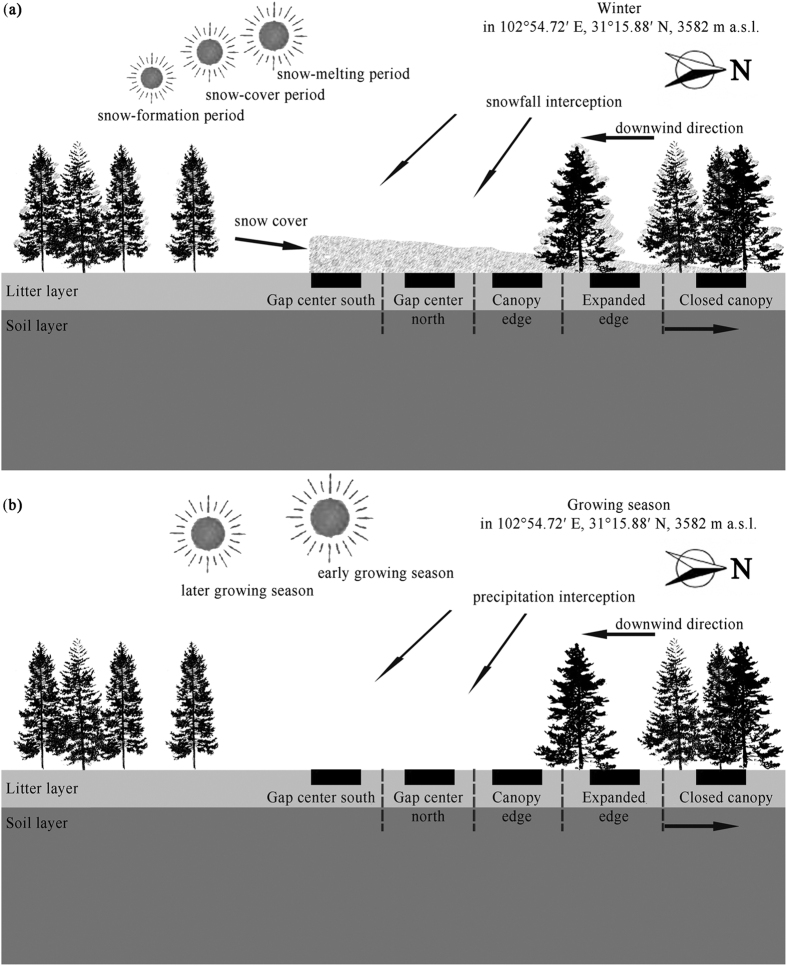
Experimental layout across landscape locations of the forest gaps in the alpine fir forest of the eastern Qinghai-Tibet Plateau (profile of forest gap). The black squares show an example of one possible spatial arrangement of litter bag placement. The relative positions and sizes of the sun represent possible temporal changes of solar radiation input in the northern hemisphere with the Earth’s revolution. During the winter, the snow cover can form early in the gap center (snow-formation period) and complete ablation can occur early below the closed canopy (snow-melting period), with complete cover throughout the snow-cover period. During the early growing season, the gap centers can receive large amounts of rainfall and sunshine, whereas strong solar radiation causes significant evaporation and decreasing soil moisture in the gap centers. Conditions during the later growing seasons were milder and more stable; i.e., there was substantial precipitation and sunlight in the gap centers.

Consistent with the hypothesis that alpine forest gaps can accelerate shrub foliar litter C, N, and P release during the cold season, the results of this study indicate that the *V*_*L*_ of C, N, and P in the two snow-formation periods, snow-cover periods, and snow-melting periods was greater in the gap centers than below the expanded canopy or closed canopy. This finding corroborates the previous findings that snow cover can accelerate litter decomposition^20^,^26^. Theoretically, thicker snow cover in the gap centers serves as an insulator (Fig. 4a) that can maintain sufficiently warm temperatures for biotic activity^15^,^27^. Consistent with findings from our earlier study (He *et al.*^9^), we found that the microbial biomass carbon (MBC) decreased from the gap center to the closed canopy as the snow-cover depth decreased during winter. This trend in the MBC among the gap locations can partly account for the release of litter C, N, and P^1^ (Table 1) during these winter periods. However, statistical analyses indicated that the MBC was not always correlated with litter C, N, and P *V*_*L*_during winter (Table 1). As the snow melts, leaching can result in the labile release of litter C, N, and P^20^. However, thicker snow cover can form early in the gap center, and ablation may occur later there^22^,^23^. The *V*_*L*_ of C, N, and P are positively correlated with the snow cover depth (SCD) (Table 1), which indicates that leaching has a greater effect on litter C, N, and P release, consistent with previous studies^20^,^28^.

### Carbon, nitrogen and phosphorus release across gap locations during the growing season

Our hypothesis that the forest gap can slow the release processes of C, N, and P in shrub foliar litter during the growing season was only partially supported by our data. Litter *V*_*L*_of C, N, and P did not follow the expected trend during the two later growing seasons, exhibiting higher values in the gap centers than in the closed canopy. The following underlying mechanisms explain the observed pattern: During the two early growing seasons, the gap centers received large amounts of rainfall and sunshine (Fig. 4b). This strong solar radiation caused significant evaporation, lowering the moisture in the gap centers. In comparison, in the expanded edge and closed canopy, evaporation was reduced because of the canopy shelter, and the higher temperature and moisture were beneficial to microbes^29^,^30^. In addition, after undergoing greater physical destruction due to frequent freeze-thaw cycles in winter (Supplementary Table S3), the litter in the expanded canopy and the closed canopy becomes more decomposable^16^,^28^. As a result, the combination of these factors produced increases in *V*_*L*_ of C, N, and P from the gap center to the closed canopy during the two early growing seasons, which is consistent with our hypothesis. Furthermore, the correlation analyses provided evidence that the average temperature was negatively correlated with the *V*_*L*_ of C, N, and P during the two early growing seasons (Table 1), indicating that ambient temperature *in situ* is a dominant factor during the early growing season in the alpine forest. The observations from the later growing seasons, which might have been due to seasonal changes^21^, contradict our hypothesis. These seasons produced mild and stable conditions (Fig. 4b, Supplementary Table S3), and sufficient precipitation and sunlight in the gap centers was favorable for decomposers^9^,^21^ and promoted the release of litter nutrients^1^,^31^. Additionally, leaching continued in the gap centers^9^,^21^. Accordingly, there was a decrease in release of litter C, N, and P from the gap center to the closed canopy.

### Seasonal effects on carbon, nitrogen and phosphorus release and stoichiometric ratios

We also observed that most of the litter C was released during the first year, particularly during the first winter, which is consistent with the findings of Wu *et al.*^16^. The presence of freshly senesced litter with more labile C components and greater availability of soluble nutrients may account for this observation^16^,^32^. Additionally, physical destruction due to freeze-thaw cycles as temperatures dropped below 0 °C directly increased the litter decomposability^26^,^33^. During winter, the thicker snow cover in the gap centers supports biotic activity and contributes to litter decomposition^15^,^27^. However, the litter below the expanded canopy and closed canopy is often exposed to extreme sub-freezing temperatures, which depresses the activity of decomposers^21^. This activity recovers during the early growing season as the temperature increases^9^, which explains why the highest *V*_*L*_ of C in the gap centers and below the canopy edge was observed during the first snow-cover period and why the highest *V*_*L*_ of C below the closed canopy was observed during the first early growing season (Fig. 2a,b). The willow litter lost more C than did the bamboo litter (Fig. 1a,b) due to the higher quality (higher N concentrations and lower C:N ratio) of the former^34^.

Findings by Moore *et al.*^35^ based on a decomposition experiment spanning 12 years in Canada indicated that litter net N loss occurred at C:N mass ratios between 33 and 68. For their early research^36^, the critical C:N ratio was 37–51 for 10 foliar litter species at 18 sites over 6 years. In our study, however, litter N and P in all locations displayed immediate absolute release (during the first snow-formation period, i.e., days 0–58; Fig. 1) and continued releasing these nutrients throughout the winter of the first year, although the initial C:N ratio of the bamboo litter (35.23) exceeded the 12-year lowest critical value. The result in this case suggests that the snowfall combined with the frequency of the freeze-thaw cycle (FFCT) and the leaching power during the phase of original litter decomposition accelerated the original litter N release prior to the biological enrichment in the alpine forest (Table 1: litter N loss was positively correlated with the FFCT during SF1). Additionally, the temperature was consistently below 0 °C (Table S3) and the soil underwent deep freezing^26^ during the winter. Litter N and P release during the winter periods may be attributed to microbes that survived by using nutrients that could be obtained only from the foliar litter rather than from the freezing soil^37^, although the soil C:N and C:P ratios were far less than those of the litter^14^.

Furthermore, the litter N and P levels in all locations and associated with both species displayed clear enrichment during the first growing season, indicating that without the stress of freezing, the chemistry of the forest floor at the site affected the nutrient release from the litter^35^,^38^. Due to early melting of the snow cover at the second snow-melting period (Fig. S1), the soil temperature approached 0 °C, and the microbial activity increased sharply after this thawing. The temperature together with the high soil moisture content likely led to anaerobic microsites and promoted denitrification^39^,^40^, resulting in enrichment during this period, as expected should occur during the early growing season, like in the first year of the experiment (Fig. 1c–f).

P mineralization is closely linked with environmental factors^35^, and higher temperatures stimulate litter P release^41^. Accordingly, combined with abundant P in the litter during the early stage of decomposition, our findings indicate that the highest *V*_*L*_of P from both types of litter in most locations occurred during the first later growing season. Compared with the initial values (Fig. 3), the foliar litter C:N ratio decreased and the C:P and N:P ratios increased with time because the ratios *in situ*^14^ caused the ratios in the litter to converge as the litter decomposed^35^. In addition, our results provide insight into the processes of litter C, N, and P release and suggest that the forest gap can accelerate litter nutrient release (Fig. 1). These shrub species release P faster than N^35^; thus, the C:N ratio increased and the C:P and N:P ratios decreased from the gap center to the closed canopy with the litter decomposition (Fig. 3).

In summary, the C, N and P release dynamics of both litter types during the 2-year study displayed clear decreasing tendencies from the gap center to the closed canopy, particularly during the two winters; the reverse was observed during the growing season. However, N and P in the litter displayed enrichment during the first early growing season and the second snow-melting period. The litter C:N, C:P, and N:P ratios were affected by the forest gap locations and the chemistry of the soil *in situ*: the litter C:N ratios increased from the gap center to the closed canopy, whereas the opposite trend was observed in the C:P and N:P ratios. The values of the pairwise ratios among C, N, and P converged as the decomposition proceeded. Accordingly, gap formation accelerates litter carbon and nutrient release; however, these processes might be limited by reduced snow cover thickness and snow cover time associated with winter warming or by the disappearance of gaps as forest regenerate. In the future, additional attention should be paid to ecological processes occurring in winter and to critical seasonal periods of litter decomposition in alpine forests.

## Materials and Methods

### Site description

The study site is located in the Miyaluo Nature Reserve (102°53′–102°57′ E, 31°14′–31°19′ N, 2458–4619 m a.s.l.), Li County, Sichuan, southwest China. The reserve is in a transitional area between the Tibet Plateau and the Sichuan Basin. The annual mean temperature ranges from 2 to 4 °C, with maximum and minimum temperatures of 23 and −18 °C, respectively. The annual precipitation is approximately 850 mm. The seasonal soil freeze-thaw period begins in early November after the first snowfall, and the soil remains frozen for 5 to 6 months^42^. The tree canopy is dominated by *Abies faxoniana* and *Sabina saltuaria*. The understory shrubs are dominated by *Salix paraplesia*, *Fargesia nitida*, *Rhododendron lapponicum*, *Berberis sargentiana*, *Sorbus rufopilosa*, *Rosa sweginzowii* and other species. The herb layer is dominated by *Cacalia spp.*, *Cystopteris montana*, *Carex spp.*, *Cyperus spp.* and other species (Supplementary Table S1). A detailed report on the soils of the reserve can be found in Zhu *et al.*^28^ and Ni *et al.*^14^.

### Experimental design

To assess the release of C, N and P from the foliar litter as a function of gap location, as study sites, we selected three circular gaps (each with a diameter of 25 m) with similar canopy structures in a representative fir forest (102°54.72′ E, 31°15.88′ N, 3582 m a.s.l.) in the Miyaluo Nature Reserve. In addition, five locations within each gap, each measuring 4 × 4 m, were distributed from the gap center to the closed canopy (gap center south, gap center north, canopy edge, expanded edge and closed canopy) along a downwind traverse at 3–4 m intervals to ensure adequate sampling of the diverse microenvironments (Fig. 4).

The nylon mesh bag technique^43^,^44^ was used to quantify the release of C, N and P from the foliar litter. In September 2010, freshly senesced leaves of dwarf bamboo (*Fargesia nitida*) and willow (*Salix paraplesia*) were collected from the floor of the forest. To avoid damaging the litter structure, we air-dried the leaves for more than two weeks at room temperature. When the litter bags were initially prepared, five samples of each litter type were oven-dried at 65 °C for 48 h to determine the ratio between the air-dried and oven-dried mass. This ratio was used to convert the initial air-dried mass of the litter to its oven-dried mass, and the subsamples were then ground (0.3-mm sieve) and analyzed to determine their initial chemical composition (Supplementary Table S2).

Each sample of air-dried litter (totaling 10 g of dry weight for each species) was placed in a nylon bag (20 × 20 cm) with 0.055-mm mesh on the bottom and 1.0-mm mesh on the surface, with the edges sealed^17^. A total of 1500 litter bags (3 gaps × 5 locations × 2 species × 10 sampling dates × 5 replicates) were placed on the forest floor from the gap center to the closed canopy on October 26, 2010. In addition, we placed an iButton DS1923-F5 Recorder (iButton DS1923-F5, Maxim/Dalls Semiconductor, Sunnyvale, USA) in one bag at each of the five sampling locations and hung one on a shrub; these devices were used to measure the temperature in the litter bags and the ambient atmosphere, respectively (Supplementary Fig. S1).

To understand how the seasonal periods affect the foliar litter C, N and P dynamic processes, we divided the winter and the growing season into the snow-formation period (SF), the snow-cover period (SC), the snow-melting period (ST), the early growing season (EG) and the later growing season (LG), and we sampled the litter bags 10 times over 2 years based on previously collected local data and field observations (Table 2). The litter bags were randomly collected from each location on each sampling date. The snow thickness was measured using a ruler on each sampling date during the winter (Supplementary Fig. S1).

### Sample analyses and calculations

After the arthropods and foreign roots were removed from the litter bags, the samples were oven-dried at 65 °C for 48 h to determine the dry mass and concentrations of C, N, and P. These concentrations were determined as described by Lu *et al.*^45^. The carbon concentration was determined using the dichromate oxidation-sulfate-ferrous titration method. Sub-samples of 0.2000 g were acid-digested with a solution of 8 ml H_2_SO_4_ (*ρ* = 1.84 g cm^−3^) and 3 ml H_2_O_2_ at 190 °C for 30 min. The digested solution was then transferred to a 100-ml volumetric flask, quantified, filtered, and stored for N and P analyses. N and P were determined by indophenol-blue colorimetry and phosphomolybdenum-yellow colorimetry, respectively. All analyses were performed in triplicate.

To characterize the temperature dynamics during each critical period, we calculated the average temperature (AT) and frequency of the freeze-thaw cycle (FFTC) from the daily mean temperatures and the number of freeze-thaw cycles per period, respectively (Supplementary Table S3):


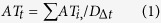



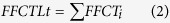


The remaining contents (*R*_*m*_) of C, N and P during each specific period of the 2-year study were calculated using the equation:


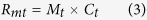


To exclude the effects of time length (day number) on the release rate during each specific period, the daily release rates (*V*_*L*_) of C, N and P during each period were calculated using the equation:


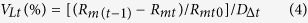


where *AT*_*i*_ is the daily mean temperature during the specified period; *D*_△*t*_ is the number of days between the specified and previous sampling dates; *FFCT*_*i*_is the daily number of freeze-thaw cycles during the specified period (a freeze-thaw cycle was defined as whenever the temperature dropped below 0 °C for at least 3 h and was followed by a rise above 0 °C for at least 3 h, and vice versa^46^); *M*_*t*_ is the litter’s remaining mass when sampled; *C*_*t*_ is the concentration of C, N or P when sampled; *R*_*m(t−1)*_and *R*_*mt*_ represent the remaining C, N or P contents between the current and previous sampling dates, respectively; and *R*_*mt0*_is the initial C, N or P content.

### Statistical analyses

An independent *t*-test with an alpha level of 0.05 was used to evaluate the differences between the initial substrates of the two species. After the pairwise comparisons tests were performed using multivariate ANOVA (MANOVA), the responses of the variables (remaining C, N, and P contents, the daily release rates and stoichiometric ratios) across the location to the decomposition time/period were evaluated by exponential regression or non-parametric LOESS regression with a 95% confidence interval (95% CI). After verifying the general ANOVA hypothesis, detailed post hoc mean comparisons of significant differences in the litter variables (all the measurements and calculations) among the locations during each decomposition period were performed using Tukey’s HSD. The homogeneity of the variances was tested using Levene’s test. Any data sets failing this test were log-transformed before further analysis to help satisfy the requirement of variance homogeneity. Univariate analysis of variance was conducted with gap locations and sampling periods serving as fixed factors (gap locations was treated as a nested factor) using the nested model of General Linear Models to examine the effects of the two factors on C, N, and P contents remaining in the foliar litter, daily release rates and stoichiometric ratios. The relationships between abiotic factors (AT and FFCT), biotic factors (MBC) and the foliar litter daily release rates of C, N, and P were determined using Pearson’s correlation coefficients. All analyses were performed using the Statistical Product and Service Solutions program (SPSS version 21.0).

## Additional Information

**How to cite this article**: He, W. *et al.* Gap locations influence the release of carbon, nitrogen and phosphorus in two shrub foliar litter in an alpine fir forest. *Sci. Rep.* **6**, 22014; doi: 10.1038/srep22014 (2016).

## Supplementary Material

Supplementary Information

## Figures and Tables

**Table 1:** Correlation coefficients (r) for the average temperatures (AT, °C), frequencies of the freeze-thaw cycle (FFCT, times), snow cover depths (SCD, cm) and microbial biomass carbon (MBC, mg/kg dry mass) with the foliar litter C (A), N (B) and P (C) daily release rates.

	(A)				(B)				(C)			
	AT	FFCT	SCD	MBC	AT	FFCT	SCD	MBC	AT	FFCT	SCD	MBC
SF1	0.670^**^	0.717^**^	0.699^**^	0.679^**^	0.54^5**^	0.600^**^	0.605^**^	0.259	0.447^*^	0.333	0.486^**^	0.143
SC1	0.682^**^	–0.016	0.624^**^	0.858^**^	0.468^**^	0.159	0.471^**^	–0.126	0.809^**^	0.159	0.813^**^	0.687^**^
ST1	0.13	–0.550^**^	0.646^**^	0.359	0.166	–0.256	0.381^*^	–0.317	–0.176	–0.604^**^	0.671^**^	0.536^**^
EG1	–0.532^**^	–0.764^**^		0.716^**^	0.347	0.524^**^		–0.267	0.792^**^	0.687^**^		–0.798^**^
LG1	0.668^**^	–0.349	0.556^**^	–0.209	0.684^**^	–0.420^*^	0.456^*^	–0.232	0.520^**^	–0.596^**^	0.666^**^	–0.513^**^
SF2	–0.139	0.052	–0.109	–0.054	0.294	0.383^*^	0.348	0.608^**^	0.288	0.296	0.355	–0.166
SC2	0.570^**^	0.464^**^	0.579^**^	0.112	0.364^*^	0.162	0.255	0.235	0.668^**^	0.498^**^	0.648^**^	0.202
ST2	0.174	–0.366^*^		0.025	–0.773^**^	0.843^**^		–0.630^**^	–0.122	0.164		0.286
EG2	–0.645^**^			0.508^**^	–0.620^**^			0.768^**^	–0.706^**^			0.612^**^
LG2	0.16	0.202		0.245	0.557^**^	0.645^**^		0.706^**^	0.361^*^	0.458^*^		0.345

Significant effects: *p < 0.05; **p < 0.01; n = 30.

**Table 2:** Sampling dates and the corresponding litter exposure days and seasonal periods.

Sample No.	1	2	3	4	5	6	7	8	9	10
Sampling date	December 23, 2010	March 3, 2011	April 19, 2011	August 19, 2011	November 8, 2011	December 27, 2011	March 7, 2012	April 28, 2012	August 25, 2012	October 29, 2012
Litter exposure day	58	128	175	297	378	427	498	550	669	734
Seasonal period	SF1	SC1	ST1	EG1	LG1	SF2	SC2	ST2	EG2	LG2

SF1, the first snow-formation period; SC1, the first snow-cover period; ST1, the first snow-melting period; EG1, the first early growing season; LG1, the first later growing season; SF2, the second snow-formation period; SC2, the second snow-cover period; ST2, the second snow-melting period; EG2, the second early growing season; LG2, the second later growing season.
