# Supplementary material for: Gap locations influence the release of carbon, nitrogen and phosphorus in two shrub foliar litter in an alpine fir forest
Source: Sci Rep. 2016 Feb 24;6:22014. doi: 10.1038/srep22014 (PMC4764935; doi:10.1038/srep22014)
Supplement: Supplementary Information [file srep22014-s1.pdf]

SUPPLEMENTARY INFORMATION FOR

Gap locations influence the release of carbon, nitrogen and phosphorus in two shrub foliar litter in an alpine fir forest

Wei He[1], Fuzhong Wu[1,2], Wanqin Yang[1,2*], Danju Zhang[1,2], Zhenfeng Xu[1,2], Bo Tan[1,2], Yeyi Zhao[1], Meta Francis Justine[1]

*Corresponding author

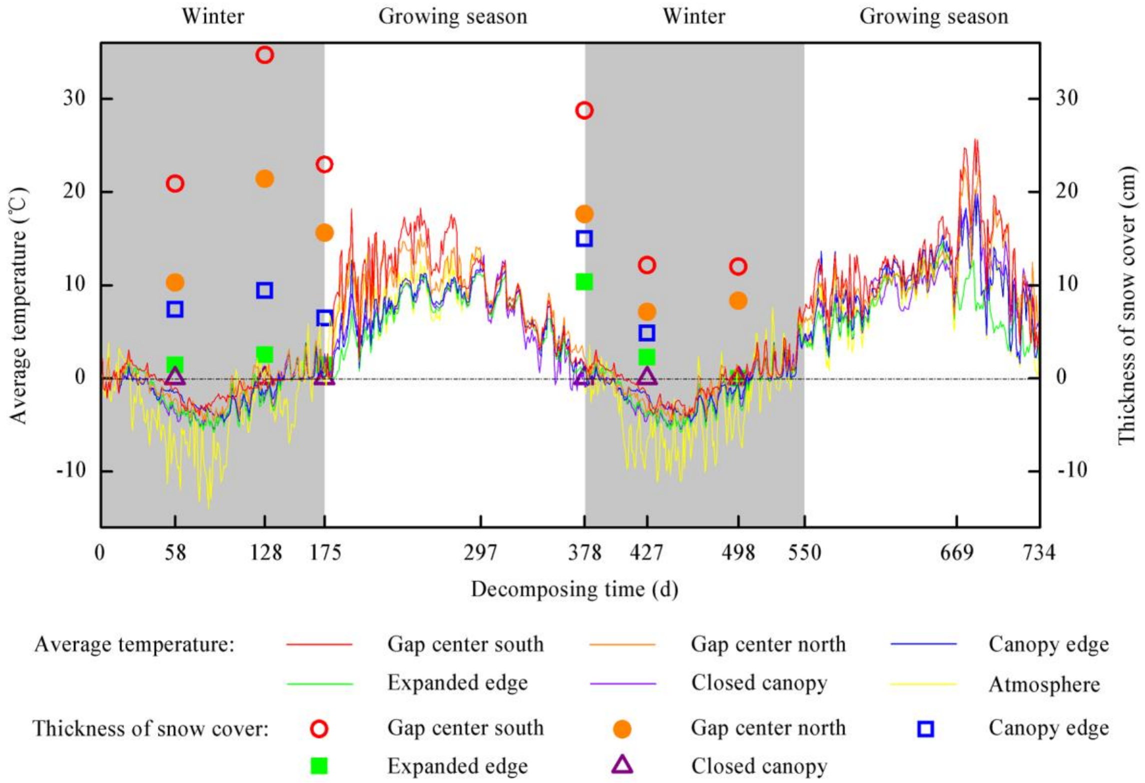

Supplementary Fig. S1 Average temperatures (lines) in the ambient foliar litter and in the atmosphere from October 26, 2010, to October 29, 2012 (734 days total) and snow depths (symbols; mean, n = 5) from the gap center to the closed canopy on each sampling date in the alpine fir forest of the eastern Qinghai-Tibet Plateau.

Supplementary Table S1 Common tree species, understory shrubs and herbs in the Miyaluo Nature Reserve

Trees	Shrubs	Herbs
Abies faxoniana	*Berberis sargentiana*	*Aconitum fangianum*
Betula albo-sinensis	*Berberis silva-taroucana*	*Ajuga ovalifolia*
Larix mastersiana	*Fargesia nitida*	*Cacalia spp.*
Sabina saltuaria	*Lonicera lanceolata*	*Carex spp.*
	Potentilla fruticosa	*Clematis spp.*
	Rhododendron delavayi	*Cyperus spp.*
	Rhododendron lapponicum	*Cystopteris montana*
	Rhododendron pachytrichum	*Deyeuxia scabrescens*
	Rhododendron taliense	*Dryopteris sinofibrillosa*
	Rosa omeiensis	*Epilobium angustifolium*
	Rosa sweginzowii	*Pedicularis roylei*
	Salix paraplesia	*Poa crymophila*
	Sorbus rufopilosa	*Sibiraea angustata*

Supplementary Table S2 Initial concentrations of C, N, and P and their pairwise ratios in shrub foliar litter of *Fargesia nitida* and *Salix paraplesia* (mean ± SD, n = 5).

Species	C (g kg^{-1})	N (g kg^{-1})	P (g kg^{-1})	C/N	C/P	N/P
Fargesia nitida	317.71 ± 16.60a	9.02 ± 0.12b	0.94 ± 0.07b	35.23 ± 1.38a	339.80 ± 9.11a	9.66 ± 0.64b
Salix paraplesia	371.89 ± 31.55a	14.33 ± 0.26a	1.28 ± 0.06a	25.93 ± 1.74b	290.72 ± 10.31b	11.23 ± 0.36a

Lowercase letters denote significant differences between species within the same variable (independent samples *t*-test, $p < 0.05$).

Supplementary Table S3 Calculated results of average temperature (AT, ℃) and frequencies of the freeze-thaw cycle (FFCT, times) from the gap center to the closed canopy during each decomposition period.

		1st year					2nd year				
Gap position		SF1	SC1	ST1	EG1	LG1	SF2	SC2	ST2	EG2	LG2
Gap center south	AT	0.38	–2.00	–0.14	12.33	6.1	0.3	–1.90	2.2	11.39	14.19
	FFTC	48	0	2	15	0	35	38	10	0	5
Gap center north	AT	–0.49	–2.40	1	10.8	6.74	–0.79	–2.13	1.82	9.76	13.45
	FFTC	49	54	23	13	0	39	59	20	0	5
Canopy edge	AT	–0.66	–2.86	0.37	7.86	6.08	–0.84	–2.79	1.04	9.98	9.97
	FFTC	41	5	18	13	0	27	11	21	0	9
Expanded edge	AT	–0.61	–3.53	0.32	6.9	5.43	–0.98	–3.35	1.09	8.48	6.43
	FFTC	38	22	34	1	0	26	28	36	0	0
Closed canopy	AT	–1.02	–3.47	0.5	7.67	5.19	–1.46	–3.24	1.2	8.41	11.93
	FFTC	39	15	38	0	27	25	21	39	0	0

SF1, the first snow-formation period; SC1, the first snow-cover period; ST1, the first snow-melting period; EG1, the first early growing season; LG1, the first later growing season; SF2, the second snow-formation period; SC2, the second snow-cover period; ST2, the second snow-melting period; EG2, the second early growing season; LG2, the second later growing season.

Supplementary Table S4 Results of two-way ANOVA of the effects of gap landscape location and sampling period on the remaining C, N, and P content.

		C				N				P			
Species	Factors	df(n)	df(error)	F	P	df(n)	df(error)	F	P	df(n)	df(error)	F	P
	Gap	4	100	220.956	<0.001	4	100	47.008	<0.001	4	100	703.546	<0.001
Fargesia nitida	Time(Gap)	45	100	128.662	<0.001	45	100	316.546	<0.001	45	100	138.906	<0.001
	Gap	4	100	248.495	<0.001	4	100	145.319	<0.001	4	100	939.519	<0.001
Salix paraplesia	Time(Gap)	45	100	218.832	<0.001	45	100	173.391	<0.001	45	100	80.484	<0.001

Supplementary Table S5 Results of two-way ANOVA of the effects of gap landscape location and sampling period on the C, N, and P daily release rates.

		C				N				P			
Species	Factors	df(n)	df(error)	F	P	df(n)	df(error)	F	P	df(n)	df(error)	F	P
	Gap	4	100	14.537	<0.001	4	100	4.224	0.003	4	100	20.597	<0.001
Fargesia nitida	Time(Gap)	45	100	17.911	<0.001	45	100	135.212	<0.001	45	100	131.733	<0.001
	Gap	4	100	15.283	<0.001	4	100	3.605	0.009	4	100	17.415	<0.001
Salix paraplesia	Time(Gap)	45	100	34.390	<0.001	45	100	244.904	<0.001	45	100	30.696	<0.001

Supplementary Table S6 Results of two-way ANOVA of the effects of gap landscape location and sampling period on the pairwise ratios among C, N, and P.

		C				N				P			
Species	Factors	df(n)	df(error)	F	P	df(n)	df(error)	F	P	df(n)	df(error)	F	P
	Gap	4	100	7.914	<0.001	4	100	127.759	<0.001	4	100	130.943	<0.001
Fargesia nitida	Time(Gap)	45	100	428.597	<0.001	45	100	64.239	<0.001	45	100	94.062	<0.001
	Gap	4	100	170.514	<0.001	4	100	235.222	<0.001	4	100	1121.545	<0.001
Salix paraplesia	Time(Gap)	45	100	340.595	<0.001	45	100	132.474	<0.001	45	100	95.522	<0.001
